# Worm sensation!

**DOI:** 10.1186/1744-8069-1-8

**Published:** 2005-02-15

**Authors:** Liam J Drew, John N Wood

**Affiliations:** 1 Molecular Nociception Group, Dept. of Biology, Medawar Building, UCL, Gower Street, London, WC1E 6BT, UK

Mechanosensation plays a pivotal role in many aspects of pain pathology, yet the mammalian molecular transduction apparatus responsible for this sensory modality remains unknown. In January's edition of *Nature Neuroscience*, O'Hagan, Chalfie and Goodman [1] have provided direct electrophysiological evidence that somatic mechanotransduction in *C. elegans *is mediated by a complex of proteins previously identified in genetic screens for impaired touch sensation. Are the homologues of these proteins important for pain sensation in mammals? Perhaps surprisingly, the balance of evidence suggests that other proteins are better candidate noxious mechanosensors in mammals.

Many forms of pain, be it in acute, inflammatory or disease-related conditions, are triggered by mechanical stimuli. However, in mammals there is very little understanding of the molecular transduction process that converts mechanical stimuli into a change in membrane excitability. Studying mechanosensation in mammals is hampered by the diffuse and inaccessible distribution of nerve terminals in the periphery. The few studies of receptor potentials, made using extracellular recordings (mainly from Pacinian corpuscles of the cat's mesentery), do however suggest that mechanical stimuli depolarise termini by directly gating cationic channels [2].

**Figure 1:**
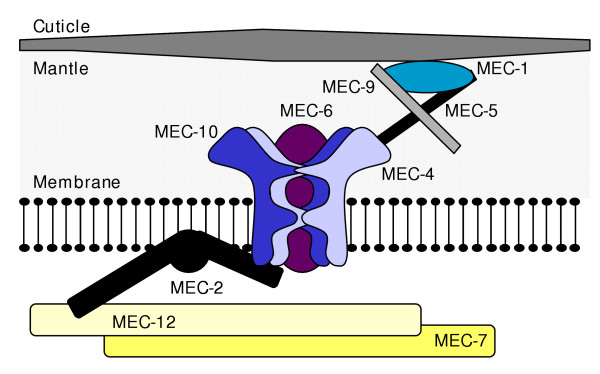
Schematic diagram of the proposed mechanotransduction complex in *C. elegans *body touch receptors. At its centre is an ion channel composed of MEC-4, 6 and 10, which interacts with the intracellular protein MEC-2. MEC-7 and 12 are microtubule proteins required for normal mechanosensation (they may be important for localisation or gating of the complex). MEC-1, 5 and 9 are extracellular proteins whose functions await further characterisation. (Figure adapted from Ref. 4.)

It is genetic studies in *C. elegans *and *Drosophila *that have driven forward our molecular understanding of mechanosensation in a number of different cell types. The best-characterised system is the body touch receptor neuron of *C. elegans*; over 2 decades, Martin Chalfie and co-workers have, on the basis of genetic mutant interactions, behavioural analysis and gene cloning, devised an elegant molecular model of transduction in these cells (see Refs. 3 and 4). In this model at least 9 proteins form a mechanotransduction complex with an ion channel at its core formed by MEC-4 and MEC-10 (members of the DEG/ENaC ion channel superfamily) and apparently MEC-6 (a paraoxonase-like protein, [5]). The complex also contains extra- and intracellular structures that the ion channel is tethered to, via specific linker proteins (probably stomatin-like MEC-2 internally, [6]), such that sheering between them gates the channel (Fig. 1). Up until the present study however, no one had recorded ionic currents attributable to activation of this complex. Now though, Chalfie, Rob O'Hagan and Miriam Goodman (a pioneer of *in situ *patch-clamping in nematodes) have measured mechanoreceptor currents (MRCs) in body touch receptors and provided direct evidence supporting the model of transduction [1].

To record from body touch receptors, O'Hagan *et al *used transgenic animals in which these cells were labelled with GFP. Using immobilised worms, the authors released the internal hydrostatic pressure away from the recording site and then exposed the cell bodies of posterior, lateral receptor neurons. Then patch-clamp recordings were made from the cell body while the mechanosensitive neurite was stimulated with a glass probe applied to the body wall. The authors observed that both the application and withdrawal of mechanical stimuli evoked rapidly adapting inward currents, whose amplitude was proportional to the magnitude of the stimulus. Consistent with the currents being mediated by members of the DEG/ENaC family, they were carried by sodium ions and blocked by amiloride. Next, given the extensive genetic analysis of mechanosensation in this species the investigators were able to extend their work by studying receptor currents in a range of mutant animals. Firstly, it was shown that null mutations in MEC-4, MEC-2 and MEC-6 abolished MRCs, suggesting that these 3 proteins (which physically interact) are essential for channel gating. An important control experiment was to show that voltage-gated currents in these mutants were normal. Subsequently, it was found that other (behaviourally less severe) mutations in MEC-4 and MEC-10 greatly reduced MRC amplitude and significantly altered the current-voltage relationship of MRCs. Hence, this is the first direct demonstration that MEC-4 and MEC-10 form the mechanotransducing ion channel in *C. elegans*. Finally, the group analysed MRCs in nematodes with a mutation in MEC-7, a β-tubulin required for formation of touch cell specific 15-protofilament microtubules, which had been hypothesised to be intracellular "anchors" required for channel gating. Interestingly, despite a large decrease in their amplitude and threshold, MRCs were not abolished in these mutants suggesting that MEC-7 is not an absolute requirement for channel gating.

This study represents a confirmation of the key aspects of a long-standing model of mechanotransduction. However, the relationship between this system and those in operation in mammalian somatic mechanosensation remains unclear. In mammals there are 9 identified DEG/ENac channels, which form two subfamilies; the epithelial sodium channels (ENaCα, β, γ and δ) and the acid sensing ion channels (ASIC1-4, and the closely related intestinal sodium channel, INaC). ENaCα, β and γ together form a constitutively active channel principally associated with non-neuronal tissues. β and γ ENaC do appear to be expressed in DRG neurons [7] but, as yet, their function there has not been studied. However, much interest was aroused in ASICs as potential mechanosensors because they are highly expressed in sensory neurons and 2 isoforms (ASIC3 and 1b) are almost exclusively expressed in these cells. Currently, the only known activator of these channels is external acidification, which gates 4 of the 6 known splice variants when they are expressed alone (interestingly MEC-4 and MEC-10 are not gated by protons) [8]. However, it has been suggested that if localised in a mechanotransduction complex analogous to that found in *C. elegans*, ASICs might mediate mammalian mechanosensation [9]. To test this hypothesis Michael Welsh and Gary Lewin collaborated in generating null mutants of ASIC1, 2 and 3 and assessing their somatosensory phenotypes using the skin-nerve preparation. In stark contrast to the dramatic effects of null mutations in MEC-4 and MEC-10, ablation of these genes had minor effects on mechanosensory responses. The first study found an approximate halving of the suprathreshold firing rates of rapidly adapting low threshold mechanoreceptors (LTMs) in ASIC2 nulls and a minor decrease in slowly adapting LTMs whilst the responses of all other fibre types were unchanged from wild type values [10]. In ASIC3 knockouts, rapidly adapting LTMs had an *increased *sensitivity to mechanical stimuli whereas Aδ-mechanonociceptors showed a decrease in responsiveness [11] and in ASIC1 null mutants cutaneous mechanosensation was unchanged from wild-type levels [12]. Whilst the analysis of double and triple knockouts would be worthwhile given the possibility that the remaining subunits functionally compensate for the missing ones in null mutants (although their expression was unchanged at the transcriptional level), the phenotypes of these animals is not consistent with ASICs being major transducers of mechanical stimuli in mammalian sensory nerves. Moreover, in an analysis of a separate line of ASIC2 nulls, no alteration in the sensitivity of rapidly adapting LTMs was found [13] and no group has reported mechanical gating of ASICs. Although mechanical gating of ion channels that are mechanosensitive *in situ *may be difficult using *in vitro *systems, different subpopulations of cultured DRG neurons are known to display distinct mechanically activated cationic currents [14] and these currents are unchanged in ASIC2 and/or 3 null mutants [15]. These data therefore suggest that other ion channels act as the primary mechanotransducers in mammals.

Whilst research on body touch receptors in *C. elegans *focussed attention on DEG/ENaC channels, genetic screens of other mechanosensory systems, particularly in *Drosophila*, have also revealed major roles for TRP channels in mechanosensation. In fruit flies, TRP-like channels NOMPC [16] and Nanchung [17] have been strongly implicated as mechanotransduction channels in Type I mechanosensors required for touch and hearing, respectively. In *Drosophila *larvae, Painless, a TRPV-like protein, is expressed in nociceptor-like cells and mutants have defective responses to noxious thermal and mechanical stimuli [18]. Also, in *C. elegans *OSM-9 is required for nose touch avoidance [19]. Research in mammalian systems has now produced evidence suggesting TRPA1 may be the transduction channel in hair cells [20] whilst TRPC1 has recently been shown to be directly mechanosensitive [21]. With regard to noxious mechanosensation, TRPV4 knockouts were found to have behavioural deficits in response to tail pressure [22], although this channel seems to be expressed at much higher levels in keratinocytes than in sensory neurons. Given that a number of TRP channels are already known to be central to thermosensation and inflammatory function in nociceptors, members of this family represent interesting candidates for mammalian noxious (and innocuous) mechanosensors.

In conclusion, the primary candidates for the role of mammalian mechanotransducers are members of the TRP and DEG/ENaC ion channel families, both of which are remarkably functionally diverse. However, the evidence supporting a function for any particular channel in mammalian mechanotransduction is much weaker than in invertebrate systems. Interestingly, the diversity of DEG/ENaC channels in *C. elegans *(28 homologues) in comparison to mammals (mice have 8) is striking, and the observation that mechanosensitive channels in nematodes form a distinct subgroup that all contain a specific extracellular regulatory domain [23] makes extrapolation of the *C. elegans *results to mammals less certain. Related to the diversity of putative mechanosensory ion channels is the issue of diversity in cellular systems that mediate mechanosensation. Despite similarities, the phylogenetic relationship between mammalian hair cells and primary somatosensory neurons and the analogous cells types in invertebrates is poorly established. Also, the extent to which chemically mediated mechanosensation functions in certain systems, potentially including some forms of mechanically induced pain, is currently unclear (for example see Ref. 24). Thus, much remains to be learnt regarding the molecular basis of mechanotransduction and when this is achieved, it should be possible to determine the evolutionary relationships of multiple mechanosensory systems. In addition, identification of the molecular basis of noxious mechanosensation should provide exciting new analgesic drug targets.
